# Amyotrophic Lateral Sclerosis: Focus on Cytoplasmic Trafficking and Proteostasis

**DOI:** 10.1007/s12035-025-04831-7

**Published:** 2025-04-03

**Authors:** Shrilaxmi MS, Saradindu Banerjee, Santosh R. D’Mello, Somasish Ghosh Dastidar

**Affiliations:** 1 Center for Molecular Neuroscience, Kasturba Medical College, Manipal, Manipal Academy of Higher Education, Manipal, Karnataka 576104 India; 2 College of Arts and Sciences, Louisiana State University, Shreveport, LA 71115 USA

**Keywords:** Neurodegenerative diseases, Amyotrophic lateral sclerosis, Endoplasmic reticulum stress, Unfolded protein response, Vesicular transport, Axonal transport

## Abstract

Amyotrophic lateral sclerosis (ALS) is a progressive and fatal motor neuron disease characterized by the pathological loss of upper and lower motor neurons. Whereas most ALS cases are caused by a combination of environmental factors and genetic susceptibility, in a relatively small proportion of cases, the disorder results from mutations in genes that are inherited. Defects in several different cellular mechanisms and processes contribute to the selective loss of motor neurons (MNs) in ALS. Prominent among these is the accumulation of aggregates of misfolded proteins or peptides which are toxic to motor neurons. These accumulating aggregates stress the ability of the endoplasmic reticulum (ER) to function normally, cause defects in the transport of proteins between the ER and Golgi, and impair the transport of RNA, proteins, and organelles, such as mitochondria, within axons and dendrites, all of which contribute to the degeneration of MNs. Although dysfunction of a variety of cellular processes combines towards the pathogenesis of ALS, in this review, we focus on recent advances concerning the involvement of defective ER stress, vesicular transport between the ER and Golgi, and axonal transport.

**Graphical Abstract:**

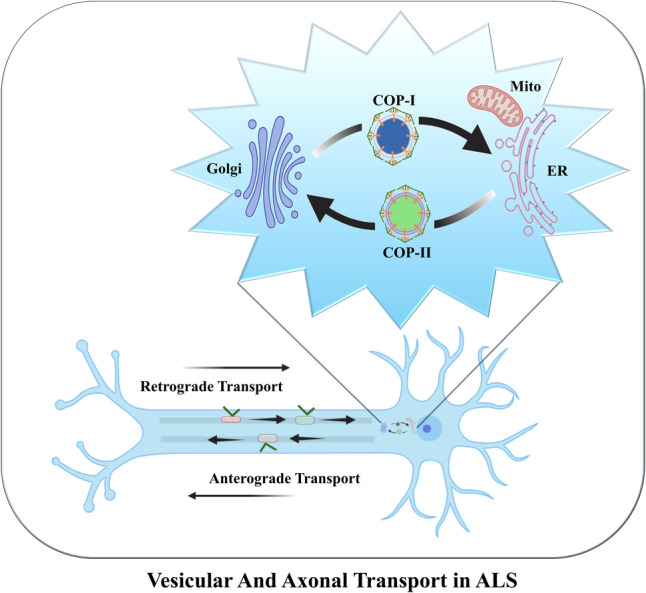

## Introduction

Amyotrophic Lateral Sclerosis (ALS), also referred to as Lou Gehrig’s disease, is an adult-onset, progressive, and fatal neuromuscular disorder characterized by the degeneration of upper and lower motor neurons (MNs) of the brain stem and spinal cord [1–5]. The average age onset of the disease is about 55–60 years, with first indications revealed as muscle weakness and difficulty in coordinating movement. These deficits, arising from muscle atrophy and MN denervation, are followed by disabilities in speaking, swallowing, respiratory paralysis, and eventually death, usually within 2—5 years after symptom onset. ALS is the most common motor neuron disease in adults, with a prevalence of approximately 5 cases per 1,00,000 individuals. About 10% of ALS cases result from inheritance in a Mendelian or non-Mendelian pattern and are referred to as familial ALS (fALS) [6, 7]. In the other ~ 90% of cases, the disease is caused by a combination of environmental factors and genetic susceptibility and is referred to as sporadic ALS (sALS) [6, 7]. Despite having distinct etiological causes, patients with fALS and sALS display indistinguishable symptoms and neuropathological features.

**Fig. 1:**
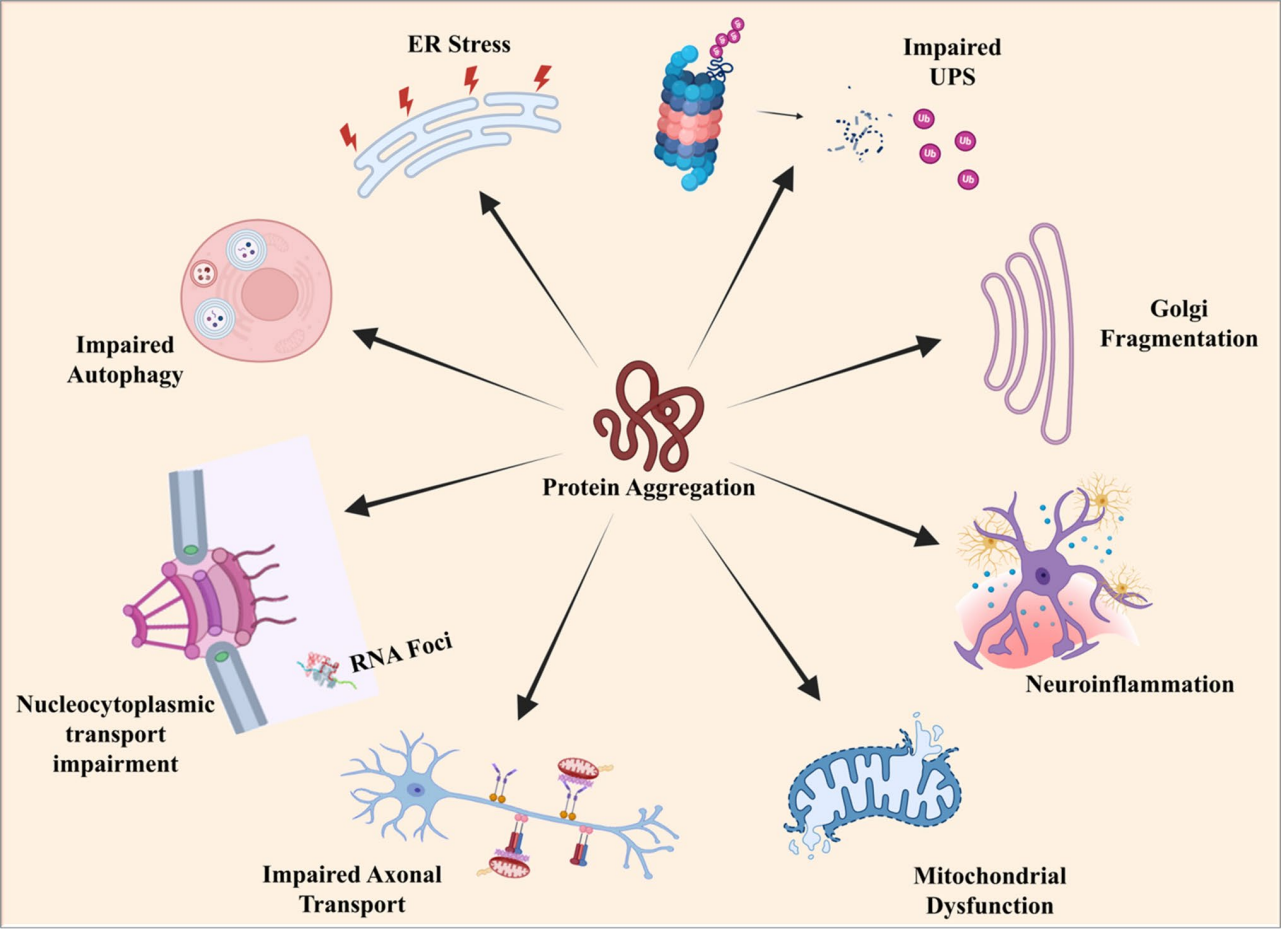
Multiple cellular processes impacted by the accumulation of protein and RNA aggregates. Accumulation of protein aggregates impairs several cellular processes including autophagy and proteasomal degradation, mitochondrial function, nucleocytoplasmic transport, and axonal transport. Additionally, protein and RNA foci in the nucleus affect nuclear function. Impaired vesicular transport between the ER and Golgi causes Golgi dysfunction and fragmentation

More than 50 genes have been identified as fALS-causing or disease-modifying genes with the most common and well-studied of the disease-causing genes encoding superoxide dismutase 1 (SOD1), TAR DNA binding protein 43 (TARDBP), Fused in sarcoma (FUS), and chromosome 9 open reading frame 72 (C9orf72) [5, 7–9]. Mutation of the C9orf72 gene represents the most common cause of fALS, accounting for about 50% of all cases [10]. SOD1 is a copper/zinc-binding superoxide dismutase that detoxifies superoxide [11], TDP43 and FUS are RNA-binding protein [12, 13], and C9orf72 is a multifunctional protein with known roles in the regulation of vesicular transport and autophagy [10] (Fig. 1).

**Fig. 2:**
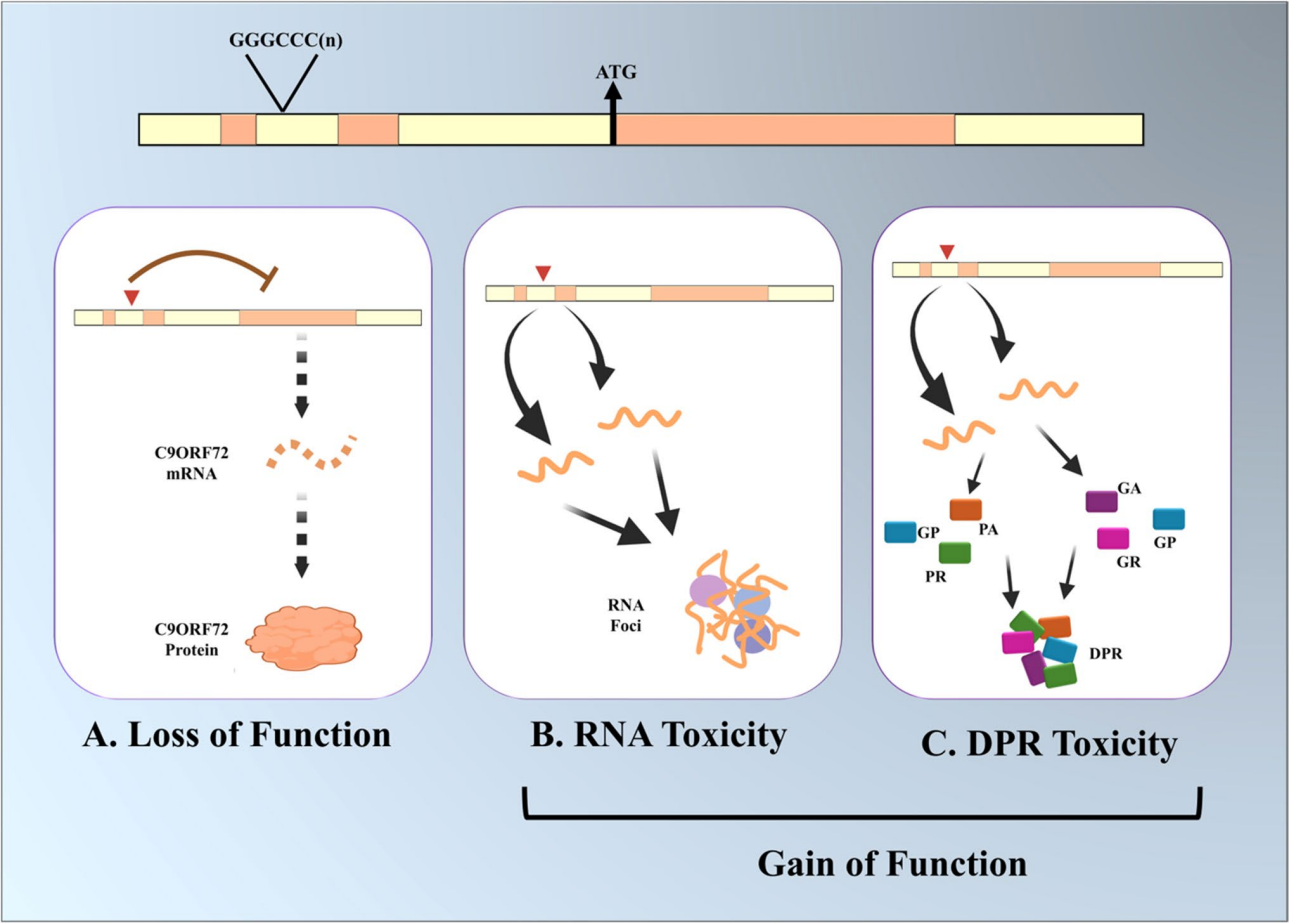
C9orf72 mutation has both loss-of-function and gain-of-function effects. Among the loss-of-function effects is reduced transcription, which leads to reduced production of functional C9orf72 protein. Gain-of-function mutations result from the production of RNA fragments that aggregate in RNA foci impairing nuclear function and the production of DPRs which aggregate in the cytoplasm impairing multiple processes including vesicular transport, axonal transport, and mitochondrial function

Studies into how ALS-linked mutations in the four major genes cause the disorder have revealed both gain-of-function and loss-of-function mechanisms [5, 8, 9, 14]. In contrast to the mutations affecting the SOD1, TARDBP, and FUS genes, which are predominantly point mutations, the C9orf72 gene mutation represents a massive hexanucleotide (GGGGCC) repeat expansion (HRE) in the first intron of the gene. The HRE region is transcribed from both strands and then translated through non-canonical repeat-associated non-AUG (RAN) translation generating five different di-peptide repeat (DPR) proteins, which then aggregate within toxic cytoplasmic inclusions [15]. In addition, the incompletely transcribed RNAs synthesized from the HRE adopt complex secondary structures and accumulate with entrapped proteins in nuclear inclusions called RNA foci [16, 17]. The absence or reduction of C9orf72 results in defects in vesicular trafficking and autophagy [18–20] (Fig. 2). Of note is that mutations of the four major genes along with the other identified ALS-linked genes together account for ~ 70% of fALS indicating that there are yet-to-be-identified disease-causing genes.

In contrast to fALS, which is inherited, sALS is believed to be caused by a combination of environmental factors and genes that confer susceptibility. The environmental factors implicated in sALS include exposure to agricultural pesticides, heavy metals, solvents, electrical magnetic fields, environmental warming, smoking, and physical exertion [21, 22]. More recently, microbiome alterations and viruses have been implicated in contributing to ALS [23–25]. A large number of genes have been identified that confer susceptibility to ALS, which may exist either as mutant or polymorphic forms [26, 27].

There are currently no effective treatments for ALS. Research aimed at gaining insight into the cellular and molecular underpinnings of ALS has utilized a variety of experimental models, including primary neuronal and cell line models, as well as in vivo models utilizing *C. elegans, Drosophila*, zebrafish, and rodents [28, 29]. Work using these models has identified many diverse cellular and molecular abnormalities that likely play a key role in the pathogenesis of ALS. Among these, the accumulation and aggregation of misfolded proteins are the best studied [1, 2, 29–31]. Other cellular abnormalities include dysregulation of RNA processing, mitochondrial dysfunction, oxidative stress, excitotoxicity, nucleocytoplasmic and vesicular transport abnormalities, Golgi fragmentation, axonal transport defects, development of cytoplasmic stress granules and other nuclear and cytoplasmic inclusions, and neuroinflammation [1, 2, 29–31]. It is noteworthy that most of these cellular defects are not unique to ALS occurring in a variety of other neurodegenerative diseases. Although which of these multiple abnormalities are disease-initiating events that directly contribute to MN degeneration in ALS (and other neurodegenerative diseases) remains to be resolved, abnormal protein aggregation connects to most of these dysfunctional processes (Fig. 1).

The protein products of all four major fALS-causing genes, SOD1, C9ORF72, TARDBP, and FUS, form aggregates in the ALS spinal cord and brain [29]. Among these, aggregates of the TDP43, the product of the TARDBP gene, is by far the most prevalent with presence in the brain stem and spinal cord of postmortem brains of almost all ALS patients, except those caused by SOD1 mutations [32], and in all sALS cases. Although TDP-43 and FUS are primarily nuclear proteins, ALS-associated protein aggregates of TDP-43 and FUS are cytoplasmic [32, 33]. In the case of C9orf72, The HRE region composed of the expanded GGGGCC repeat is transcribed from both strands and then translated through non-canonical repeat-associated non-AUG (RAN) translation generating five different di-peptide repeat (DPR) proteins—polyGA (glycine-alanine), polyGP (glycine-proline), polyGR (glycine-arginine), polyPA (proline-alanine) and polyPR (proline-arginine) – which form toxic cytoplasmic inclusions [15] (Fig. 2). Of note is that the DPRs show differences in distribution within brain regions and in abundance, with polyGP and polyGA being the most abundant [34, 35]. In addition to the production of DPRs, the incompletely transcribed RNAs synthesized from the HRE adopt complex secondary structures and accumulate with entrapped proteins in nuclear inclusions called RNA foci, which disrupt nuclear functions [16, 17] (Fig. 2).

Key to the accumulation of protein aggregates is dysfunction of protein degradation mechanisms within the cell, including the ubiquitin–proteasome system (UPS), which degrades proteins after they are enzymatically polyubiquitinated, and the autophagy machinery, which degrades proteins, other macromolecules, and dysfunctional organelles in the lysosome. Three types of autophagy have been characterized [36, 37]. In macroautophagy, cellular material, including entire organelles, is enclosed within an ER-derived double-membrane vesicle called the autophagosome, which fuses with the lysosome, resulting in the digestion of the cargo. In contrast to the “bulk degradation” in macro-apoptosis, chaperone-mediated autophagy is selective, involving the recognition of the misfolded protein by heat shock cognate 70 (HSC70), which binds to it and facilitates its entry into the lysosome through Lamp2a receptors. In microautophagy, cytosolic material is engulfed directly by the lysosome following an invagination of lysosomal membrane and encapsulation. Although generally a form of bulk and non-selective degradation, subtypes of macroautophagy that utilize autophagosomes, but which enclose and transport specific types of protein aggregates and organelles, such as mitochondria or peroxisomes, to lysosomes have been described, and are referred to as selective autophagy [38–41].

Several recent studies have described cell-to-cell transmission of misfolded protein aggregates and DPRs in ALS along neuroanatomical tracts [42, 43]. In the case of SOD1 and TDP43, the misfolded proteins can seed the misfolding of normal forms of these proteins within the cells that receive them, in a prion-like manner [42, 43]. Multiple mechanisms have been proposed for the intracellular transfer of ALS-related pathogenic proteins, including extravesicular (EV) secretion, axonal transport, synaptic transmission, and micropinocytosis. Of these, most attention has been placed on EVs, which normally transport proteins, lipids, and various RNA species between cells located in the vicinity or at a distance. EVs have been found to contain ALS-related protein aggregates and can exert cytotoxic effects on cells exposed to them. Indeed, exosomal TDP43 has been proposed to serve as a biomarker of disease progression in ALS patients [44].

Although motor neurons are the primary targets in ALS, emerging evidence suggests that alterations in GABAergic interneurons, astrocytes, and oligodendrocytes are also involved in disease pathogenesis [45, 46]. For example, astrocytes and astrocyte-conditioned medium from fALS / sALS patients and mutant SOD1 mice are toxic to wild-type MNs in vivo and in vitro culture, respectively, while conditioned medium from wildtype astrocytes protect MNs from SOD1-ALS mice [47–50]. While MNs degenerate in ALS, neurons of the frontotemporal cortex are affected in a disorder in which genetics, symptoms, and pathology are a combination of ALS and frontotemporal dementia (FTD), a neurodegenerative disorder referred to as ALS-FTD [46, 51]. As in the case of ALS, C9orf72 expansion accounts for a majority of cases of ALS-FTD [16, 52, 53]. TDP-43 aggregates are found in most patients with ALS-FTD and ~ 50% of patients with FTD [54, 55]. FUS aggregates are also found in subsets of FTD and ALS-FTD patients [56]. Despite these genetic and neuropathological similarities, however, patients with ALS-FTD are characterized by cognitive and behavioural impairments [57].

Several reviews have been written on the molecular and cellular abnormalities in ALS focusing on all these disrupted processes or focusing on specific ones. In this review, we focus on protein aggregation and specifically, the cause of protein aggregation and the effect that these aggregates have on vesicular transport between the ER and Golgi and axonal transport.

### Within the ER

**Fig. 3:**
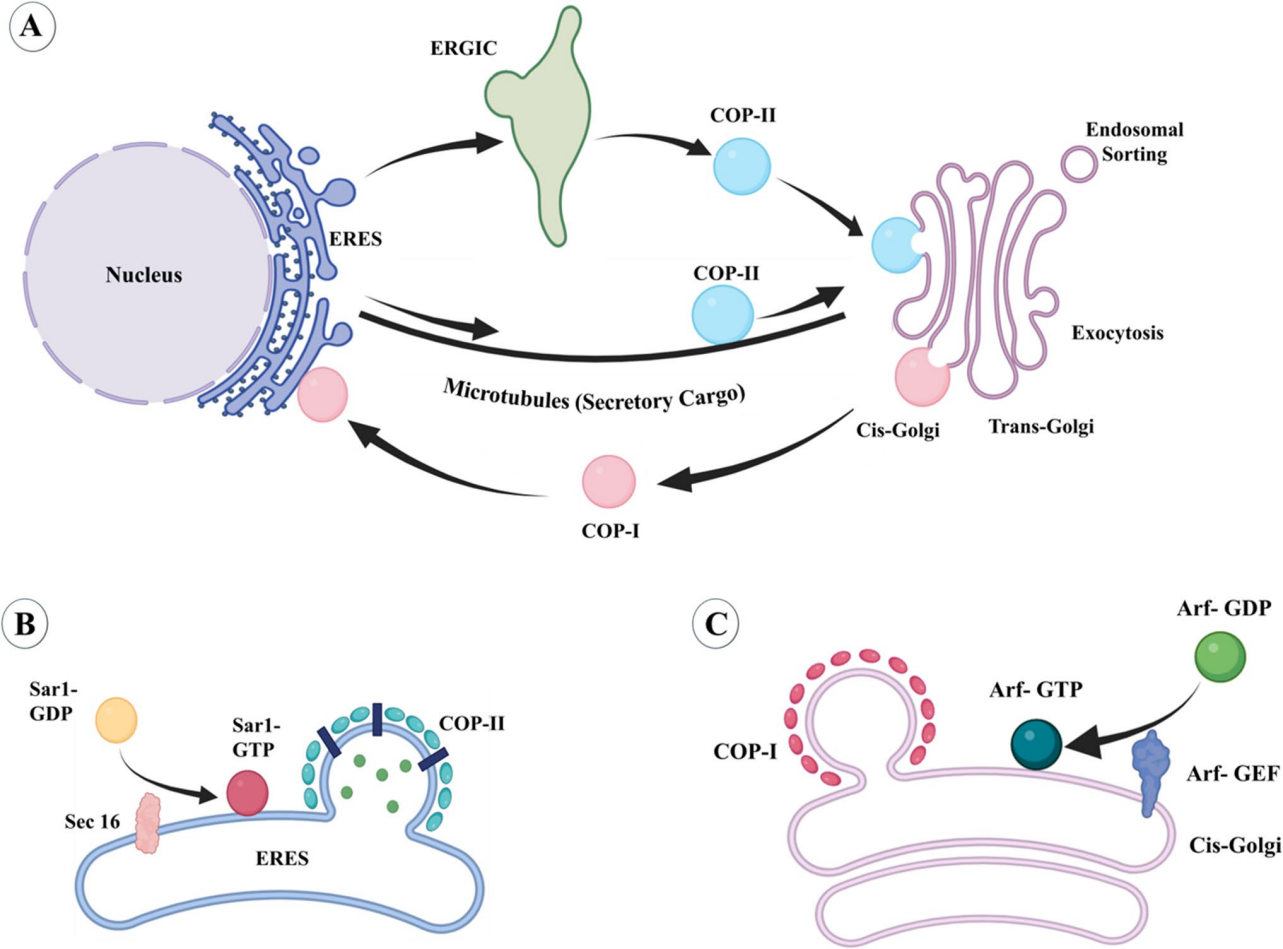
Transport between the ER and Golgi. **A** Luminal and membrane proteins from the ER are transported in COP-II vesicles which bud off from regions of the smooth ER called ERES. Most of the vesicles move the ERGIC before budding off from it and fusing to the cis-Golgi membrane. Some secretory proteins are transported in COPII-coated vesicles directly to the Golgi via microtubules. Membrane and soluble proteins are transported from the Golgi to the ER, including ER-resident proteins, that are transported in COPI-coated vesicles. **B** The vesical formation, COPII coating, and exit from the ER are initiated by the recruitment of the Sar1-GTPase to the ER membrane and activation of Sar1-GDP to Sar1-GTP by the SAR1-GEF, Sec16. **C** The vesical formation, COPI coating, and exit from the cis-Golgi are initiated by the recruitment of the Arf1-GTPase to the Golgi membrane and activation of Arf1-GDP to Arf1-GTP by the Arf-GEF

Membrane proteins, resident proteins of most cellular organelles and compartments, and proteins that are secreted by the cell are synthesized at the wall of the rough endoplasmic reticulum (ER) and are inserted into the ER through the Sec61 translocator (Fig. 3). While entering the ER, and following entry, the nascent proteins are folded and modified by chaperones and a multitude of enzymes such that they are functional [58, 59]. Properly folded membrane and soluble proteins are packaged in vesicles coated with multi-subunit COP-II proteins that bud out of the smooth ER membrane. This process is initiated by recruitment of the small GTPase, Sar1 (secretion-associated RAS-related 1 by its cognate guanine nucleotide exchange factor (GEF), Sec12. After budding and exiting from the ER membrane, COPII-coated versicles travel to and fuse with the ER–Golgi intermediate compartment (ERGIC) (Fig. 3). Many secreted proteins are transported in vesicles by microtubule-associated motor proteins [60, 61]. From ERGIC or by transportation via microtubules, COP-II vesicles fuse with the walls of the cis-Golgi where the proteins are further modified, sorted, and then transported within vesicles to their destination.

**Fig. 4:**
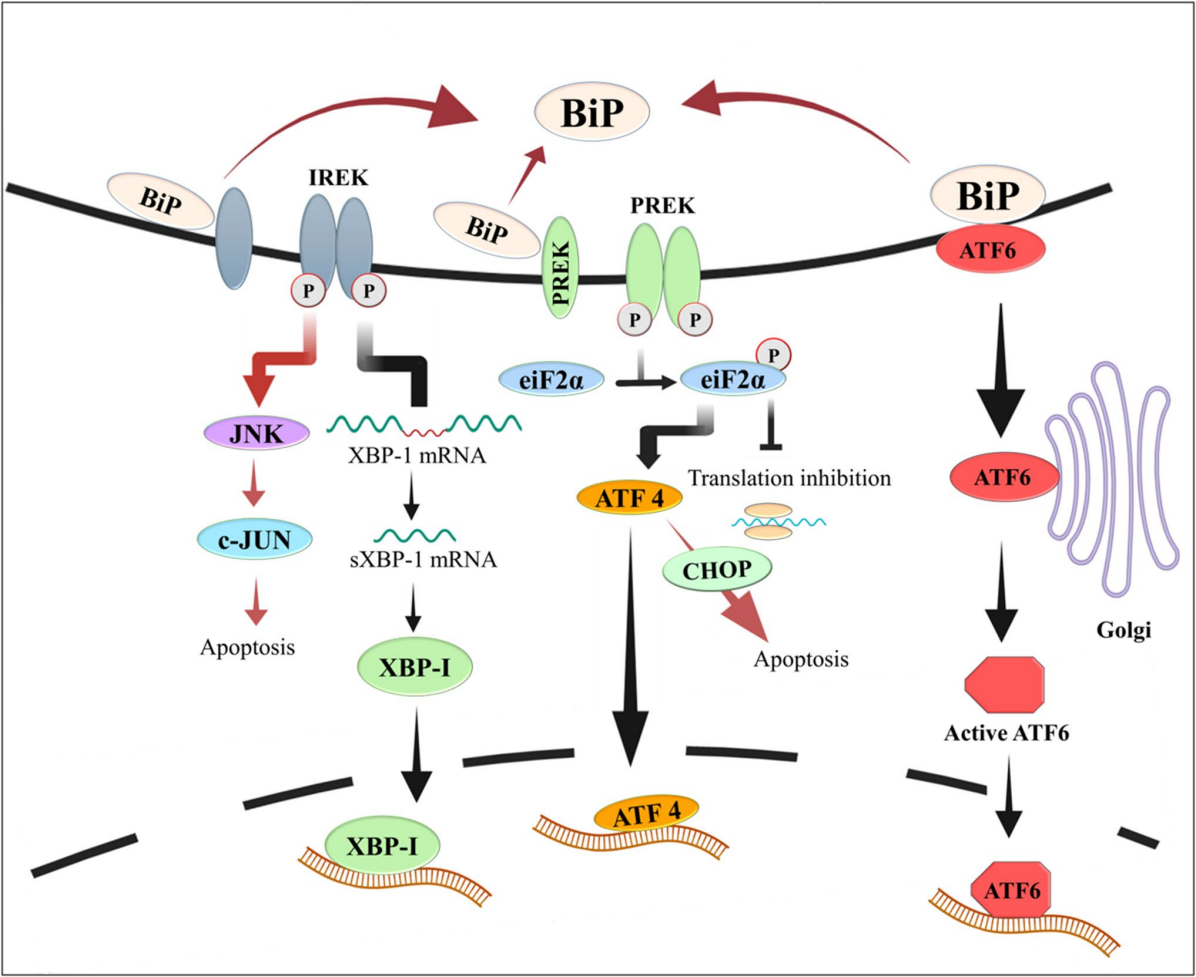
The UPR signalling pathways. When the ER is functioning under normal conditions, the UPR composed of the IRE-1a, PERK, and ATF6 is inhibited by the binding of BiP. Under conditions of ER stress, BiP disassociates activating the catalytic activities of IRE1α and PERK and permitting the translocation of ATF6 to the Golgi apparatus, where it is cleaved to generate active ATF6. Together, these proteins upregulate the production of chaperones, strengthen ERAD, and upregulate lipid synthesis for expansion of the ER membrane. If the stress cannot be resolved, the IRE1α and PERK pathways activate apoptosis-promoting molecules, including JNK and ATF4 (which now stimulate the transcription of pro-apoptotic genes). Figure modified from Lukas et al. *Adv. Med. Sciences* (2019)

A large proportion of the newly synthesized proteins entering the ER do not fold properly. Chaperones within the ER attempt to re-fold them. But if unsuccessful, the improperly folded proteins are transported out of the ER, ubiquitinated, and degraded by cytoplasmic proteasomes through a process called ER-associated degradation (ERAD). In addition to ERAD, misfolded or aggregated proteins are degraded in the lysosome through autophagy. The prolonged build-up of misfolded proteins in the ER that cannot be resolved by ERAD or autophagy causes “ER stress”, which triggers the activation of specific intracellular signalling pathways, collectively referred to as the unfolded protein response (UPR) [62–64] (Fig. 4). The UPR relieves ER stress by expanding the ER membrane thus increasing lumen space, inhibiting new protein synthesis to reduce further stress, increasing the expression of chaperones in the ER to refold the misfolded protein(s), and upregulation of the ERAD machinery for enhanced disposal of the misfolded proteins. If the UPR is unable to relieve ER stress, it activates apoptosis-promoting genes, resulting in the demise of the cell [65]. The three major signalling pathways comprising the UPR are activated through three distinct proteins, IRE1α (inositol-requiring protein-1α), PERK (protein kinase RNA (PKR)-like ER kinase), and ATF6 (activating transcription factor 6) [62–64] (Fig. 4). When the ER is not stressed, these UPR-initiating proteins are kept inactive through association with BiP (binding immunoglobulin protein), a molecular chaperone also referred to as GRP-78. Under conditions of ER stress, BiP/ GRP78 releases IRE-1α, PERK, and ATF6, permitting them to signal (Fig. 4).

Activation of IRE-1α signalling results in the splicing of X-box-binding protein 1 (XBP1) mRNA, generating sXBP1 mRNA, which encodes a transcription factor, XBP1s. XBP1s stimulate the expression of genes that encode chaperones and that increase ERAD activity [66]. IRE-1α activation can also lead to excessive endoribonuclease activity, which causes mRNA degradation at the ER membrane resulting in reduced protein translation, a mechanism referred to as regulated IRE1-dependent decay (RIDD) [67]. The PERK pathway reduces protein synthesis by inhibiting eF2 activity through phosphorylation of eIF2α, a component of the eIF2 complex. Although global translation is inhibited by PERK/phospho-eF2α-mediated inhibition of translation, several mRNAs escape inhibitions by using upstream open reading frames (uORFs) or an internal ribosome entry site (IRES). Among these mRNAs are those encoding activating transcription factor 4 (ATF4) and ATF5 [68, 69], which function during ER stress to stimulate transcription of other genes that are also resistant to translation inhibition by PERK and are involved in reducing stress, re-initiation of global cap-dependent translation, or inducing apoptosis. The third arm of the UPR is activated by translocation of ATF6 to the Golgi where it is cleaved at the membrane, releasing its cytosolic portion which functions as a transcription factor to activate chaperone genes and lipid biosynthesis (Fig. 4).

Besides being a component of the UPR, PERK is part of a broader cellular stress response, termed the integrated stress response (ISR), that consists of three other kinases, which function to regain cellular homeostasis or promote cell death [70–72]. These are protein kinase R (PKR), which is activated by viral infection; heme-regulated eIF2α kinase (HRI), which is activated by hypoxia, and general control nonderepressible 2 kinase (GCN2), which is activated by amino acid deprivation. Although triggered by distinct dyshomeostatic signals, the pathways activated by all four ISR kinases lead to the inhibition of global translation through phosphorylation of eIF2α. In addition to the UPR and ISR, ER stress can be alleviated through ER-specific autophagy receptors that can promote lysosomal degradation of the misfolded proteins or, if the ER becomes dysfunctional, activate ER-phagy. ER-phagy is a form of selective macro-autophagy that specifically packages dysfunctional ER into autophagosomes for lysosomal degradation [73]. Unresolved ER stress resulting from the accumulation of misfolded proteins affects other organelles. One of these is the mitochondria with which the ER makes direct contact through multi-protein structures called mitochondria-endoplasmic reticulum (ER) contacts (MERCs) [74]. Communication between the ER and mitochondria is particularly important in the management of calcium homeostasis in the ER. Depletion of calcium from the ER contributes to protein aggregation, ER stress, and apoptosis triggered by excessive ER stress [75]. Besides affecting ER functions, impairment of MERCs affects a variety of mitochondrial activities including maintenance of calcium homeostasis, lipid exchange, detection of metabolic alterations, mitochondrial division, and mitophagy [74].

The consistent presence of protein aggregates both in animal models of ALS and in the brain and the spinal cord of patients indicates that dysfunction of protein folding mechanisms and/or defective protein clearance mechanisms are involved in disease pathogenesis [76–79]. Among the large number of proteins responsible for the proper folding of nascent proteins in the ER is a family of over twenty protein disulfide isomerases (PDIs) [80, 81], which possess both chaperone and oxidoreductase activity. Overexpression of ERp57, a member of the PDI family (also referred to as PDIA3), is protective in a transgenic mouse model of mutant SOD1-ALS and inhibits mis localization, aggregation, and the toxic effects of mutant TDP-43 in MN-like cell lines [82–84]. Interestingly, missense and intronic variants of PDI and ERp57 are associated with motor dysfunction and ALS [85–87]. It is noteworthy that, unlike other PDI proteins that are localized in the ER and associated with the ER membrane, ERp57 localizes in the lumen of the ER and also in other subcellular compartments, including the cytoplasm, mitochondria, and nucleus where other functions have been proposed [88–91]. The extent to which impaired functions in the non-ER compartments contribute to ALS pathogenesis is unclear. In the mitochondria, ERp57 modulates calcium uptake [88]. Dysfunction of calcium homeostasis in the mitochondria in ALS MNs is well-documented [92–94].

The cytoplasmic inclusions of accumulated proteins in ALS are ubiquitin-positive, further indicating failure of the proteasome to degrade them. Indeed, several genes, variants of which cause or contribute to ALS, are involved in proteasomal or autophagic protein degradation, including C9orfF72 [17, 53, 95, 96], Optineurin (OPTN) [97, 98], Valosin-Containing Protein (VCP) [99], vesicle-associated membrane protein B (VAPB), TANK binding kinase (TBK1), and UBQLN2 [8, 100]. Although mutation of C9orfF72 can promote MN degeneration through a gain-of-function manner, C9orf72 is a key mediator of autophagic degradation of proteins acting at multiple levels. Loss of C9orf72 function, or C9orf72 haploinsufficiency, results in multiple defects related to autophagy, including lysosomal dysfunction, reduced autophagic flux, and accumulation of p62, an autophagy receptor within aggregates [10, 17, 101, 102]. It is noteworthy. however, a recent study that examined these processes using iPSC-derived MNs from C9orf72-ALS patients found insignificant changes [103], suggesting the need for additional analyses to resolve these issues. UBQLN2 is a protein that associates with other proteins on the outer membrane of the ER to mediate ER-Golgi vesicular transport as well as transport of ubiquitinated proteins to the proteasome for degradation. ALS-associated mutations of UBQLN2 impair ERAD [104] and ER-Golgi transport, which results in the fragmentation of ERGIC and Golgi, and consequently, ER stress [105]. VCP, also referred to as p97, is a ubiquitously expressed AAA+ ATPase required for both proteasomal degradation and autophagy, processes mediated through the assembly of multi-protein complexes [106]. In the ERAD mechanism, VCP functions to unfold ubiquitinated proteins for proteasomal entry and degradation [107]. In the autophagic mechanism, VCP functions to degrade damaged lysosomes in a process called lysophagy, in the absence of which autophagy is rendered dysfunctional [108, 109]. Both these degradative functions of VCP are impaired by ALS-associated mutations [106]. A single mutation in the VAPB gene is a rare cause of fALS in certain geographical regions of the world but not others [110]. VAPB, as well as VCP, are part of MERCs, which tether mitochondria to the ER [111]. Expression of ALS-associated mutant VAPB causes MERC dysfunction, leading to disrupted calcium transports between the ER and mitochondria, a pathological alteration that has been well-described in ALS [111]. Mutant VAPB-induced calcium dyshomeostasis leads to mitochondrial and ER dysfunction and consequently, of mitochondria, OPTN is an autophagy receptor that is involved in the clearance of cytoplasmic protein aggregates and dysfunctional mitochondria through direct binding [112]. Not only do ALS-associated mutations of OPTN affect the clearance of protein aggregates but may also play a causal role in the formation of these aggregates in ALS [112]. TBK1, a member of the IKB kinase (IKK) family, has been identified as an essential cargo recruitment regulator functioning through p62 and OPTN phosphorylation in autophagy and mitophagy. The impairment of TBK1 results in autophagy deregulation and contributes to protein aggregation in ALS pathophysiology [113]. Moreover, defective autophagy caused by impaired TBK1 function promotes neuroinflammation [113, 114], an abnormality known to contribute to neuronal death in ALS and other neurodegenerative disease. However, one study has identified an ALS-associated TBK1 mutation that causes autophagic dysfunction and MN degeneration in mice without promoting neuroinflammation [115], suggesting that the regulation of TBK1 on the regulation of autophagy and neuroinflammation involves separate signalling mechanisms.

Although neurodegeneration in ALS primarily affects MNs, the accumulation of protein aggregates in ALS is not limited to MNs. Some evidence suggests that while other neuronal types can cope with the aggregates, a lower capacity of MNs to handle ER stress might explain why they selectively degenerate in ALS. Supporting this is the finding that motor neurons from ALS patient-derived iPSCs are more vulnerable to dying in response to chemically induced ER stress than non-motor neuron cells. Evidence of a dysfunctional ER stress response has been described in both experimental models of ALS and motor neurons derived from patient iPSCs [116]. In cultured cells, mutant SOD1, but not wild-type SOD1, induces ER stress, resulting in the aberrant activation of IRE-1 and PERK [117]. PERK haploinsufficiency in a SOD1-ALS mouse line accelerates disease onset and lifespan, suggesting the involvement of aberrant ISR in ALS pathogenesis [118]. However, this finding was disputed in a subsequent study using five different lines of mutant SOD1-ALS mice that neither haploinsufficiency of PERK nor deficiency of GADD34, a protein induced by ATF4 during the ISR and that stimulates autophagy [119], ameliorates neuropathology or disease course [120].

The selective effect of mutant SODI in inducing ER stress can be explained by its ability to interact and inhibit Derlin, a component of a multi-protein ER membrane complex that exports misfolded proteins out of the ER as part of ERAD [117]. Besides inhibiting ERAD, which causes ER stress, mutant SOD1 activates apoptosis signal-regulating kinase 1 (ASK1), an apoptosis-promoting protein [121], which is activated by the UPR and contributes to the death of the stressed cell. Expression of CHOP, another apoptosis-promoting protein and a downstream target of ASK1, is elevated both in patients with SOD1-ALS and sporadic ALS [122] and in SOD1-ALS mice [122, 123]. Expression of ATF3 and phosphorylation of c-Jun, both apoptosis mediators, is also increased in ALS mice, and this occurs before the death of MNs [123].

Like PERK, the IRE-1α and ATF6 arms of the UPR are mis regulated and contribute to ALS pathology [124]. The phosphorylation of eIF2α and the expression of XBP1 and ATF6 mRNA and protein are elevated in transgenic mutant SOD1-G93A mice and NSC34 cells, an MN-like cell line [125]. Similarly, expression, proteolytic activation, and nuclear localization of ATF6 are increased in MNs and NSC34 MN-like cell lines after expression of mutant SOD1, but not wild-type SOD1 [125]. However, another study published more recently using the same SOD1-ALS mouse model found that ATF6 expression in the spinal cord of mutant mice was not higher than in wild-type mice. Moreover, this group described that administration of repaglinide, a pharmacological inhibitor that inhibits the interaction of ATF6 with a neuronal calcium sensor, DREAM, reduced MN loss in SOD1-ALS mice, an action that was associated with increased ATF6 processing in the spinal cord [126]. The role of deregulated ATF6 processing in ALS pathogenesis has not been evaluated.

The activity of the IRE1α-XBP1 pathway leading to increased XBP1 mRNA splicing and generation of the XBP1s transcription factor is elevated in MNs from SOD1-G93A mice compared with normal MNs [125]. Splicing of XBP1 is increased on MN-like cell lines and in the spinal cord (but not cerebellum) of symptomatic SOD1-ALS mice [124, 125, 127]. Supporting a pathogenic contribution for XBP1 mRNA in ALS is the finding that SOD1-ALS mice lacking the XBP-1 gene display reduced mutant SOD1 aggregates and are more resistant to developing ALS-related pathological decline [125]. This protective effect is due to a massive increase in autophagy in mice lacking the XBP1 gene, which results in enhanced clearance of mutant SOD1 aggregates [125].

Although generally a protective cellular mechanism, several lines of evidence indicate that aberrant activation of the ISR contributes to ALS pathogenesis. Elevated ISR selectively increases RAN translation of the C9orf72 hexanucleotide repeat to produce toxic DPRs [128], an alteration that involves phosphorylated-eIF2α-dependent stress granule formation and inhibition of global protein translation [128]. Chemically induced ISR activation causes accumulation of DPRs in iPSCs-derived MNs from C9orf72ALS patients. Once produced, the accumulation of DPRs itself stimulates the ISR, resulting in a positive feedback type of pathogenic cycle. ISR activation can also be induced by RNA transcribed from the C9orf72 hexanucleotide repeat region, which adopts a substantial secondary structure [129]. Critical for the pathogenic cycle is the action of PERK. Supporting this possibility is the finding that pharmacologic inhibition of PERK protects against DPR aggregation and neurotoxicity in both fly and mouse models of C9orf72-ALS [130].

Although it is well-established that mutant forms of SOD1misfold and form aggregates, the mechanism by which wild-type SOD1 promotes sALS has not been resolved. Interestingly, when oxidized, wild-type SOD1 adopts the same misfolded conformation as mutant SOD1 [131]. Furthermore, oxidized wild-type SOD1 is present in spinal cord MNs and activates similar neurotoxic pathways as mutant SOD1 [131]. Separate studies have described that, in transgenic mice, wild-type SOD1 can heteromerize with mutant SOD1, increasing its aggregation and toxicity [132, 133]. Surprisingly and in contrast to mice, in cell culture models of SOD1-ALS, rather than promoting aggregation of the mutant SOD1, wild-type SOD1 inhibits aggregation [134]. This suggests that the stimulatory effect of wild-type SOD1 on the aggregation of mutant SOD1 requires extrinsic factors and/or other cell types, such as glial cells, that are not present in the in vitro models used for such studies.

### ER to the Golgi

The exit of COPII-coated vesicles containing membrane and soluble proteins and lipids from the ER occurs at specific locations called ER exit sites (ERES). Vesicle budding and coat assembly start with the recruitment of the GTPase, SAR1-GTP, to the ERES through the action of its GEF, SEC12, an integral ER membrane protein [58, 59]. The absence of C9orf72 leads to reduced localization of SEC12 at the ERES resulting in reduced activation of SAR1-GTPase thus impairing the assembly of COPII [135]. This impairment particularly affects lipid transport and homeostasis.

Hydrolysis of SAR1-GTP to SAR1-GDP promotes the exit of cargo-containing COP-II-coated vesicles from where they traffic to the ERGIC and then to the Golgi [58, 59]. ALS-causing mutant forms of SOD1, FUS, and TDP43 all inhibit ER to ERGIC trafficking but act by distinct mechanisms [136]. For example, mutant FUS impairs transport by acting within the ER, whereas mutant TDP-43 acts at the cytoplasmic side of the ER membrane. In contrast to mutant FUS and TDP-43, mutant SOD1 inhibits transport from the ERGIC to the Golgi. Despite these differences, all these mutant proteins disrupt RAB1 function [136]. In primary MN models of ALS produced by overexpression of mutant forms of SOD1, FUS, and TDP43, overexpression of RAB1 restores normal ER-Golgi transport [137]. RAB1 overexpression also reduces ER stress and SOD1 aggregation that occurs in response to mutant SOD1, FUS, and TDP43 expression. In spinal cord MNs of ALS patients, Rab1 mis localizes to inclusions, rendering it unfunctional [136].

### Golgi to ER

The Golgi consists of flattened membrane stacks, known as cisternae, composed of three functional compartments. The cis-Golgi is the compartment facing the ER serving to receive COP-II-coated vesicles from the ER as well as for exiting of COP-I-coated transport vesicles from the Golgi to the ER containing transport proteins, which include the vesicle-coating COP protein complexes and proteins that regulate vesicle budding and fusion, as well as proteins that are inadvertently transported to the Golgi. Much of the modification and sorting of proteins takes place in the medial Golgi, and the trans-Golgi is the compartment from which vesicles containing modified proteins exit as part of the endosomal or secretory pathways [138]. Tethering and fusion of vesicles from the ER with the cis-Golgi membrane is mediated by RAB1 and many proteins [58, 59, 138]. The structure of Golgi is maintained by a plethora of proteins, including microtubule proteins, a family of structural proteins called golgins, and transport proteins [139, 140]. Besides the “somatic” Golgi present in all cells, in neurons, additional and disconnected Golgi compartments, referred to as “outposts”, are present in dendrites and dendritic spines, and in axons, where they play a role in the trafficking of locally synthesized proteins, in local translation, and dendritic morphology through effects on microtubules [141–145]. In a *Drosophila* model of C9orf72-ALS, the number of Golgi outposts and dendritic branching is reduced, an effect attributed to the production of DPR, specifically GR and PR [146].

The retrieval of ER-resident proteins and other vesicular transport-associated proteins from the ERGIC or Golgi back to the ER requires the small GTPase ADP-ribosylation factor 1 (Arf1). Once liberated, the COP-I complex coating these vesicles disassembles to permit for the fusion of the vesicles with the membrane of the ER. The disassembly of the COP-I coat is driven through hydrolysis of Arf1-GTP by the Arf1-GTPase Activating Protein (Arf-GAP). In cell culture and *Drosophila* models of C9orf72-ALS, the RNA from the C9orf72 HRE as well as the DPR produced inhibit Arf-GAP disrupting trafficking of the Golgi-derived vesicles to the ER. While this is a gain-of-function effect, at least two studies have reported that C9orf72 can also act as an Arf-GAP an activity involving association with other proteins. C9orf72 haploinsufficiency was found to impair vesicle trafficking by functioning as an Arf-GAP [19]. However, this study was performed in the dopaminergic SH-SY5Y cell line and its significance to motor neurons or ALS pathogenesis requires further investigation.

C9orf72 possesses a DENN (differentially expressed in normal and neoplastic cells) domain, a domain present in RAB-GEFs that allows their interaction with RAB-GTPases [147, 148]. This suggests that C9orf72 could modulate the function of RAB-GTPases through direct interaction. Indeed, the interaction of C9orf72 with multiple RAB proteins has been experimentally confirmed [76]. Through such associations C9orf72 regulates autophagy and endosomal trafficking, processes that are affected by loss of Corf72 function [136]. Other studies have described that C9orf72, as part of a protein complex, can function as a RAB-GEF and promote autophagy initiation [149, 150]. C9orf72-containing complexes have also been found to exhibit ARF1-GAP activity [151, 152]. Besides being required for the disassembly of the COP-I coat, ARF1-GAP functions in the formation of nascent vesicles that are subsequently coated with the COP-I complex [153]. Loss of C9orf72 function would, therefore, reduce COP-I-vesicle production. Although best known for its function in vesicle trafficking, reduced COP-I function in neurons can have broader effects, including the reduction of MERCS leading to mitochondrial dysfunction causing calcium dysregulation, abnormal mitophagy, and reduced respiratory function [154], abnormalities that lead to ER dysfunction and neuronal degeneration. Whether C9orf72 haploinsufficiency affects mitochondrial function through its ARF-GAP activity and, consequently, COP-I function is not known. Dysfunction of MERCs and mitochondrial deterioration occur in ALS [111].

Impaired budding of COP-I-coated vesicles from the Golgi or fusion with the ER membrane can cause Golgi fragmentation [155–157]. Fragmentation of Golgi is observed in experimental models of ALS occurring before neuromuscular dysfunction and accumulation of aggregated protein inclusions, suggesting that it is an early event in disease pathogenesis [158–162]. It has been proposed that defective ER – Golgi vesicular trafficking induced by ALS-causing mutant proteins can damage motoneurons by affecting a variety of processes, including local synthesis of proteins, trafficking of proteins, autophagy, and axonal and dendritic functions [158]. Caspase-2, a caspase capable of inducing cell death, is present predominantly in the Golgi and nucleus and is activated by Golgi stress [163, 164]. Cleavage of golgins by caspase-2 can lead to a loss of Golgi architecture and cell death by apoptosis. Inhibition of caspase-2 delays golgin cleavage supporting the proposal that the Golgi participates in cell death [164]. Given that Golgi fragmentation is a common neuropathological feature of ALS, these findings suggest that signals from the Golgi, including caspase-2 activation, may trigger the death of MNs, leading to motor deficits.

### Axonal Transport – Soma to Axon Terminal and Back

Axonal transport, a process unique to neurons, ensures movement and spatiotemporal distribution of membrane-bound vesicles, proteins, lipids, mRNA, and organelles between the cell body and axon terminals [165]. Given the critical function it serves, disruption of axonal transport impacts the functioning and viability of neurons and is a common feature in many neurodegenerative diseases, including ALS [165, 166]. Because of their long axons, MNs are particularly susceptible to disruption of axonal transport. Axonal transport is mediated by motor proteins that use microtubules for the movement of cargo. The motor proteins belong to two major families, kinesins, which move cargo from the soma towards the axon terminal, referred to as anterograde transport, and dyneins, which transport cargo from the terminal towards the soma, referred to as retrograde transport [166, 167]. In contrast to dyneins, which belong to a single family of proteins, kinesis comprises a superfamily of 45 members that are further subdivided into 15 sub-families. Both kinesins and dyneins are normally autoinhibited, requiring activation, adaptors, and involvement of several other proteins to function. Axonal transport is regulated by posttranslational modification of tubulins, motor proteins, and other associated transport proteins [168, 169].

Impairment of axonal transport is regarded as a hallmark of ALS with a large body of evidence indicating that perturbation of axonal transport precedes the onset of ALS-related symptoms across different mouse models of the disease [170–173]. Early evidence of axonal transport defects in ALS was provided by electron microscopy-based neuropathological studies [174–176]. Several other pieces of more recent and compelling evidence to support a causal role for perturbed axonal transport in ALS pathogenesis. GWAS analysis has identified the KIF5A kinesin gene as an fALS-causing gene [177]. The ALS-linked KIF5A mutation promotes the death of motor neurons through a gain-of-function mechanism that results from a loss of its auto-inhibition property [178].

Genetic analysis in humans has identified a mutation in the dynein gene DYNC1H1 as likely to cause ALS [179]. The mutation affects the ability of DYNC1H1 to interact with proteins, which is necessary for its retrograde transport function. One set of proteins critically important for the microtubule motor activity of dynein is its cofactor, the dynactin complex, which acts as an adaptor to connect cargos with dynein and microtubules for retrograde vesicle transport. Mutations in the dynactin DCTN1 gene are linked to both fALS and ALS [180, 181], particularly in Caucasian populations [182].

Research conducted using multiple cell cultures and animal models of mutant SOD1-ALS has confirmed that defective anterograde and retrograde axonal transport, including transport of mitochondria, is a key abnormality in ALS [170, 183–189]. Expression of the ALS-linked mutant KIF5A in cultured cortical neurons leads to the inhibition of wild-type KIF5A through a dominant-negative mechanism leading to increased transport [178]. Mutant KIF5A has a propensity to oligomerize and aggregate, which increases the movement of the mutant protein on microtubules compared with wild-type KIF5A [190]. The tendency to aggregate and highly increased movement on microtubules has also been described in patient-derived iPSCs [191]. Supporting a causal of axonal transport in ALS is the finding that expression of mutant KIF5A in *Drosophila* results in the development of inclusions in the soma and axons and leads to MN death [192]. Mutant SOD1 impairs the anterograde transport of acetylcholine esterase and acetylcholine release from microtubules by entrapping kinesin-associated protein 3 (KAP3), a protein necessary for transport, in mutant SOD1 aggregates [189]. Overexpression of KAP3 overcomes the impairment resulting from the sequestration of KAP3 by mutant SOD1. Another mechanism causing impairment of axonal transport by mutant SOD1 is through the activation of p38 MAP kinase, which then inhibits kinesin-1 [184]. Supporting a role for aberrant p38 MAP kinase in disruption of axonal transport in ALS is the finding that the expression of three different ALS-linked mutant forms FUS, but not wild type FUS, disrupt fast transport in the squid axon by activating p38 MAP kinase [193]. Furthermore, GP-DPRs cause disruption of axonal transport and axonal degeneration, an action involving p38 MAP kinase [194]. Interestingly, other DPRs did not impair axonal transport, although GR- and PR-DPRs disrupted nuclear membrane integrity [194]. Defective transport of endosomes might be of particular significance to ALS pathogenesis. Inhibition of p38 MAP kinase rescues the defective retrograde transport of endosomes in mutant SOD1 mice [183]. In cultured MNs, the defective transport of mitochondria by mutant SOD1 results from the downregulation of Miro, a Rho GTPase 1, which is a critical regulator of mitochondrial transport in response to mitochondrial damage [188]. Disruption of retrograde transport by mutant SOD1 has also been attributed to abnormal interaction between it and dynein [195, 196]. Yet another alteration that disrupts axonal transport in SOD1-ALS involves CRMP-4 (collapsin response mediator protein-4), a member of the 5-member CRMP family of phosphoproteins that are substrates of CDK5 and GSK3β [197]. A missense mutation of CRMP4 is linked to ALS [198]. Expression of CRMP4 is elevated in the spinal cord of SOD1-ALS mice, contributing to increased binding to dynein-dynactin [197]. The heightened interaction impairs retrograde axonal transport and leads to death in MN death both in mice and in iPSC-derived neurons [197]. Expression of the ALS-associated mutant form of CRMP4 causes degeneration of MNs while disrupting its association with dynein-dynactin, which protects SOD1-ALS MNs [197, 198]. In addition to defects within MNs, cell non-autonomous mechanisms contribute to axonal transport defects in SOD1-ALS MNs resulting in exposure of normal MNs to a conditioned medium from skeletal muscle cells expressing mutant SODI disrupts mitochondrial axonal transport [199]. One exogenous factor that regulates axonal transport is BDNF [200–202], acting through the activation of axonal ERK12 [203]. While muscle-derived BDNF stimulates axonal transport of signalling endosomes, mutant SOD1 mice and MNs cultured from these mice are unable to respond to muscle-derived BDNF even though the amount of BDNF that is released is normal in mutant ALS mice [204]. The inability to respond to BDNF has been attributed to an upregulation of truncated TrkB and p75NTR receptors, which bind BDNF but cannot promote pro-survival signalling [204]*.*

In neurons, the translation of proteins can take place in axon and dendritic terminals. Such local translation depends on efficient transport and localization of mRNAs produced in the soma to distal axonal and dendritic locations [205, 206]. For their transportation in axons and dendrites, mRNA is part of messenger ribonucleoprotein (mRNP) granules which also contain RNA-binding proteins, ribosomes, and translation proteins [205]. TDP-43 is part of the mRNA-transporting mRNP granule and serves to regulate both the transport and the translation of the mRNA in dendritic spines [207]. Transport of mRNA granules is impaired both in *Drosophila* MNs and in cultured mammalian neurons and consequently, local protein translation in axon terminals is disrupted by mutant TDP-43 aggregates [208–210]. While TDP-43 moves normally in MN axons, mutant TDP43 aggregates in axons lead to defects in axonal structure and function [209, 211]. In mouse cortical neurons expression of mutant TDP-43 produces several of the cellular abnormalities of ALS, including impairment of axonal translation [212, 213]. In TDP-43-ALS mice, the extent of neuropathology and motor impairment caused by accumulation of TDP-43 aggregates in axons is similar in hemizygous and homozygous TDP-43-ALS mice suggesting that once of certain amount of mutant-TDP-43 accumulates in axons, additional accumulation has no additional effect on axonal transport [214]. iPSC-derived motor neurons from human ALS patients also display axonal TDP-43 aggregates which impairs mitochondrial transport [215]. In these MNs, inhibition of histone deacetylase-6 (HDAC6), which is a tubulin deacetylase, restores normal transport. In one mouse model of TDP-43, TDP-43^M337V^, in which ALS-associated symptoms are displayed, axonal transport is impaired, but the mice do not suffer MN death, suggesting that defective axonal transport may by itself not cause neuromuscular symptoms in TDP-43-ALS [172, 214]. In this mutant mouse line, cytoplasmic or axonal TDP-43 aggregates are absent, although the mice exhibit motor function deficit suggesting that the mutant TDP-43 aggregates are not essential for axonal transport and neuromuscular deficits. A similar conclusion was reached in another study using a different TDP-43^M337V^ mouse model [216]. Like TDP-43, FUS plays an important role in mRNA localization, transport, and local translation within axon dendrites, and dysfunction in these roles within MNs contributes to the pathogenesis of ALS [217]. FUS localizes to synapses but this is abnormal in ALS, affecting the organization of proteins in the synapse resulting in synaptic defects [218–220]. In *Drosophila* models of ALS, these mutant FUS-induced defects in synaptic organization and function lead to MN degeneration [221]. Interestingly, retrograde transport of signalling endosomes is unaffected in at least one lone of FUS-43-ALS mice (FUS^Δ14/+^ mice), although there is neuromuscular pathology and shortened lifespan [172].

Although displaying no change in morphology, electrical properties, or total mRNA level, MNs generated from human C9orf72-ALS-derived iPSCs display impaired axonal transport [222, 223]. The impairment is mediated by HER-translated PR and GR DPRs, which are associated with motor complexes and tubulin tails within microtubules [223]. Treatment of cultured iPSC-derived neurons from normal individuals or MNs in *Drosophila *in vivo with the DPRs recapitulates the defective axonal trafficking. Interestingly, sensory neurons derived from the C9orf72-ALS iPSCs also displayed axonal transport defects, although the two neuronal types displayed different transcriptomic patterns that were appropriate for their cellular identities [222]. Despite compelling evidence to the contrary, this finding suggests that axonal transport defects may not be the primary triggers of the highly selective degeneration of MNs in ALS.

In sum, whereas the evidence that disruption of axonal and dendritic transport is an initiating factor in ALS pathogenesis is reasonably convincing, a large number of molecules have been described to be involved, and more work is needed to distil the information into an integrated model of the mechanism(s) causing the disruption.

### Connections to Other Neurodegenerative Diseases

As described above, both fALS and sALS are caused by the dysfunction of specific proteins, including SOD1, TDP-43, FUS, and C9orf72 which leads to a broad spectrum of cellular abnormalities, including ER stress, the deficit in vesicular and non-vesicular transport between the ER and Golgi, and impaired axonal transport. Although with distinct symptoms and aetiologies, other age-associated neurodegenerative disorders share these and most of the other major cellular abnormalities displayed in ALS, such as nucleocytoplasmic transport defects, mitochondrial dysfunction, excitotoxicity, and neuroinflammation. How the actions of distinct disease-initiating molecular triggers converge on common cellular impairments remains to be resolved. It is noteworthy, though, that emerging evidence suggests that the molecular triggers for the development of different neurodegenerative diseases may not be as distinct as previously believed. Indeed, accumulating evidence of overlap in the molecular and cellular underpinnings of different neurodegenerative diseases has been described. For example, results from the past decade suggest that proteins contributing to or causing ALS, are also involved in abnormalities associated with other neurodegenerative diseases. For example, close to 60% of patients with AD show TDP-43 inclusions which in some cases is associated with neurofibrillary tangles, a pathological hallmark of AD [224–227]. AD patients displaying TDP-43 neuropathology display more brain atrophy and cognitive deficits than ones without it [225, 226]. TDP-43 deficiency and pathology are also observed in other diseases, including Pick’s disease, Lewy body dementia and related disorders, Huntington’s disease, and other polyglutamine diseases [228–232]. TDP-43 associate with ataxin-2, the mutation of which causes spinocerebellar ataxia, and this association enhances TDP-43 pathology [233, 234, 234–236]. Similarly, SOD1 [237–239], FUS [240–244], and C9orf72 [245–250] mutations or dysfunction are associated with neurodegenerative disorders other than ALS and ALS-FTD [245–251]. Similarly, growing evidence suggests that the aggregation of proteins that characterize other neurodegenerative diseases, such as tau (AD and other tauopathies) and α-synuclein (PD and other synucleinopathies), contribute to ALS pathogenesis [252–255]. Disruption of nucleocytoplasmic transport resulting from the r]sequestration of nucleoporins and decreased nucleocytoplasmic RAN-GTP gradient has been observed in C9orf72-ALS, AD, and Huntington’s disease [256, 257]. Aβ toxicity in vitro and AD mice results in reduced mitochondrial trafficking, an aberration caused by reduced levels of KIF2A, the mutation of which causes ALS [258]. Alteration in KIF5A-mediated axonal transport has been described in PD [259, 260], Lewy body dementia [261], and Charcot-Marie tooth neuropathy, a genetic peripheral neuropathic disorder [262–264]. PDI dysfunction, which contributes to ER stress in ALS, also participates in the pathogenesis of other neurodegenerative diseases [265–270]. Together, these and many other findings reveal crosstalk in the pathogenic signalling mechanism that leads to distinct neurodegenerative diseases.

### Other Genes Associated With ALS Implicated in Defective Protein Clearance and Cytoplasmic Trafficking

In addition to the genes encoding SOD1, FUS, TDP-43, and C9orf72, mutations or variations in several other genes functioning in vesicular, and axonal transport and protein homeostasis have been linked to ALS. For some of these genes, such as TUBA4A, Profinin-1, and PRPH, the effect of the mutations on cytoplasmic trafficking is mediated through dysfunction in cytoskeleton organization and dynamics. A brief description of some of these genes is provided below:

**KIF5A (Kinesin family member 5A):** KIF5A is a motor protein that mediates anterograde transport and axon maintenance. It is believed that KIF5A mutations, particularly a 27-exon skip-over, contribute to ALS through a loss of autoinhibition, resulting in dysregulated motor activity and impaired transport of vital cargoes, including RNA granules and mitochondria [178, 190, 271]. Expression of mutant KIF5A in *Drosophila* causes locomotor deficits and morphological changes at neuromuscular junctions with the development of toxic aggregates within axons and cell body, abnormal distribution of synaptic vesicles and mitochondria, and death of motor neurons [178, 191, 192].

**VAPB (VAP-associated protein B):** VAPB is a member of the family of ER-resident proteins, called vesicle-associated membrane-protein-associated proteins [272, 273], which functions in ER-Golgi transport, vesicle trafficking, and membrane transport [274, 275]. Within the CNS, VAPB is highly expressed in MNs [276]. While multiple rare mutations in the VAPB gene are linked to ALS, P56S is the most common, causing a dominantly inherited form of the disease, called ALS8 [274, 275]. Mutant VAPB is non-functional but entraps normal VAPs in tubular aggregates within inclusions [276]. Furthermore, ALS-linked mutations affect its interaction with other proteins and disrupt the ER structure and function [277, 278]. VAPB inclusions also occur when ALS is caused by mutations in SOD1, TARBP, and C9orf72 [274, 275]. Accumulating evidence suggests that VAPB dysfunction may be involved in sALS pathogenesis [274, 275].

**Optineurin (OPTN):** OPTN is a highly expressed and evolutionarily protein in the CNS that plays an important role in vesicular trafficking pathways and autophagy, primarily by serving as an adaptor or scaffolding protein [112, 279]. Among the proteins that bind to OPTN are the Rab8 GTPase, myosin VI, and Tank-binding kinase-1 (TBK1), the dysfunction of which has been linked to ALS [112, 279]. Mutations in OPTN are linked to both fALS and sALS [97, 98, 112, 280, 281]. OPTN-containing inclusions have been identified in patients with ALS caused by a mutation in the SOD1, FUS, TARDP, and C9orf72 genes, suggesting a central role for defective OPTN in ALS pathogenesis [112]. OPTN stimulates autophagic clearance of protein aggregates and mitochondria by acting as an autophagy receptor [112, 282]. OPTN is recruited to damaged mitochondria by PINK1-Parkin and is activated through phosphorylation by TBK1, a kinase dysfunction which is also linked to ALS [282, 283]. While OPTN function in mitophagy depends on phosphorylation by TBK1, the activation of TBK1 is dependent, at least in part, on OPTN1 [283–285]. ALS-linked mutant forms of OPTN display inefficient binding to target proteins, resulting in impaired autophagic clearance of protein aggregates and mitophagy [286, 287]. Vesicular and autophagosome trafficking by OPTN involves interaction with myosin VI, a transport protein that colocalizes with OPTN on the surface of Golgi and the plasma membrane [288–290]. When expressed in MN-like cells, ALS-linked OPTN mutations impair the association of optineurin with myosin VI, leading to defective secretory protein trafficking, ER stress, Golgi fragmentation, and impaired secretory protein trafficking [288]. Spinal cord tissue from sALS patients display reduced binding of OPTN with myosin VI in motor neurons. OPTN mutations also disrupt interaction with Rab8, an association that promotes OPTN-mediated vesicular transport between the ER and Golgi [288].

**Ubiquilin-2 (UBQLN2):** Through its interactions with E3 ubiquitin ligases and proteasomal components, UBQLN2 plays a key role in UPS-mediated protein clearance [291, 292]. Additionally, UBQLN2 participates in autophagic protein degradation of protein and mitophagy by mechanisms including maintaining acidification of autophagosomes and interaction with autophagic-regulatory molecules [292–294]. Mutations in the UBQLN2 gene have been linked to X-linked ALS [295, 296] and mice expressing ALS-linked mutant UBQLN2, but the normal protein displays disrupted proteostasis along with inclusions and motor neuron disease [297, 298]. However, overexpression of normal UBQLN in rats also promotes motor neuron death suggesting that the effect of the mutant protein is through a gain-of-function mechanism. Impaired proteasomal assembly precedes motor neuron death in rats overexpressing mutant UBQLN2 [299]. ALS-linked mutations in UBQLN2 impede both proteasomal and autophagic protein clearance [300]. Inclusions containing UBQLN2 and TDP43 are present in the CNS of ALS patients with UBQLN2 mutations, but also in patients with both sALS and fALS without UBQLN2 mutations [301]. ALS-linked mutations of UBQLN2 impair ER to Golgi transport, which results in ER stress and dysfunction, and Golgi fragmentation [105]. Analysis in vitro has revealed that UBQLN2 and OPTN2, another protein the mutation of which is linked to ALS, colocalize to the specific endocytic vesicles [302]. ALS-linked mutations of either of the two proteins lead to disruption of the vesicular colocalization resulting in impaired autophagy and the formation of inclusions [302].

**TBK1 (Tank-binding protein-1):** Although initially identified and studied based on its role in immunity signalling, more recent work has demonstrated the roles of TBK1 in the regulation of autophagy, including mitophagy [113, 114]. Through interactions with other proteins, such as OPTN (see above), and phosphorylation of adaptor proteins, TBK1 plays a critical role in the recruitment of cargo for autophagic clearance [113]. Mutations, generally non-sense or frameshift, of TBK1 cause fALS or increase the risk of ALS through reduced expression, and consequently function [303–307]. Activation of TANK is particularly important during proteostatic stress [113, 303]. It is believed that these mutations may cause ALS through impairment of autophagy and mitophagy, resulting in the accumulation of toxic aggregates and damaged mitochondria. TBK1 activity is reduced in the spinal cord of patients with sALS [303] suggesting impaired upstream signalling. Proteostatic stress-dependent activation of TBK1 involves mitochondria-associated membrane (MAM), the contact site between the mitochondria and ER [303], suggesting that impairment in the structure or function of mitochondria/MAM would impair TBK1 activation.

**SIGMAR1 [Sigma-1 receptor (σ1R)]:** SIGMAR1 is a chaperone protein located within MAMs [308–310]. It is highly expressed in MNs, and involved in a variety of important cellular processes, including protein folding, ER organization, ER-mitochondria signalling, mitochondrial function, and autophagy [308–310]. Not surprisingly, SIGMAR1 function is considered essential for neuronal survival, and protection against neurodegeneration [308, 309]. Mutation in the SIGMAR1 gene causes ALS (as well as other MN diseases) [311–314]. Mice expressing ALS-causing mutant SIGMAR1 develop an ALS-like pathology, including MN degeneration. SIGMAR1 knockout mice exhibit locomotor deficits and exacerbate the disease phenotype of mutant SOD1-ALS mice [315, 316]. Interestingly, while SIGMAR1 knockout mice display MN degeneration, sensory neurons are unaffected [317]. Analysis of MNs cultured from knockout mice reveals defects in axonal transport, mitochondrial dysfunction, and MAM collapse [317]. MAM collapse is a known feature of ALS caused by SIGMA1 mutations, but also other fALS-causing genes and in sALS [313, 318, 319]. Levels of SIGMAR1 are reduced in the spinal cord of sALS patients and abnormally localized with MNs [320]. Interestingly, agonists of SIGMAR1 have been identified and described to be protective against MN degeneration in both in vitro and ALS mice [321, 322].

**SQSTM1 (Sequestome-1):** The SQSTMI gene encodes p62, an autophagy receptor that functions in selective autophagy, a process in which proteins or organelles are selectively sequestered within an autophagosome through the actions of selective autophagy receptors [39, 323, 324]. p62 has also been found to link autophagy to the UPS, suggesting a broader role in protein clearance [324]. Multiple mutations and variations in the SQSTM1 gene are associated with ALS [325–329]. Besides facilitating the clearance of protein aggregates and mitophagy, p62 is recruited to damaged lysosomes and is involved in their clearance through lysophagy [330]. Inclusions containing p62 aggregates in spinal cord motor neurons of patients are a consistent feature of sALS and a lower burden of these aggregates is associated with longer survival [331].

**FIG4 (Factor-Induced Gene 4):** FIG4 is a phosphoinositide 5-phosphatase that is involved in controlling the turnover of PI(3,5)P2, a low abundance signalling lipid present in endolysosomes and involved in the regulation of vesicles transport to the trans-Golgi [332]. FIG4 is also involved in regulating endosomal to lysosome function, an action that is independent of its catalytic activity [333]. FIG4 deficiency has been described to cause a lysosomal dysfunction and two different lysosomal storage disorders affecting the nervous system [334]. Multiple studies have identified ALS-causing mutations in the FIG4 gene, some of which cause an aggressive form of the disease [335–339], likely through a loss of function mechanism

**VCP (Valosin-Containing Protein or p97):** VCP, also called p97, is an evolutionarily conserved, ubiquitously-expressed, and multifunctional AAA + ATPase that, along with adopting a hexameric oligomer and through association with a host of cooperating partners, regulates cellular homeostasis through the regulation of ER-associated degradation, endolysosomal sorting, disassembly of stress granules, and clearance of damaged lysosomes by autophagy [109, 340–343]. In these various functions, VCP functions to unfold protein substrate (generally ubiquitinated) by threading them through the central pore of its hexameric oligomer [344, 345]. Work in cultured cells and patient iPSC-derived MN indicate that mutations in VCP cause fALS likely by affecting its diverse functions [106, 342, 343].

**CHMP2B (Charged Multivesicular Body Protein 2B):** CHMP2B is an evolutionarily conserved protein widely expressed in the brain and is an essential subunit for the endosomal sorting complex required for transport III (ESCRT-III) [346]. Along with the other ESCRT complexes, ESCRT controls the biogenesis of multicellular vesicular bodies [347]. In addition, CHMP2B plays roles in endosomal sorting and autophagic degradation [346]. ALS-causing missense and deletion mutations in the CHMP2B gene have been identified [346, 348–350]. Expression of an ALS-causing mutant form of CHMP2B, which contains a deletion within intron 5 of the gene, CHMP2B^intron5^, causes hyperphosphorylation and aggregation of TDP43 [351]. On the other hand, the knockdown of CHMP2B reduces the TDP43 toxicity in *Drosophila* and mammalian ALS models [351] suggesting that the mutation has a gain-of-function effect. Expression of normal levels of mutant CHMP2B results in increased interaction with ERCRT II proteins leading to defective synaptic vesicle trafficking and cycling [352, 353]. Furthermore, mutant CHMP2B hinders RAB7 endosomal recruitment and causes defects in retrograde transport mechanisms [354].

**NEFH (Neurofilament, Heavy-Chain Polypeptide):** NEFH plays a key role in the formation of the neuronal cytoskeleton, which contributes to the maintenance of axonal structure and enables axonal transport. Variants in the NEFH gene increase the risk of sALS and may also represent a cause of the disease [355–359]. Interestingly, a NEFH intronic tetranucleotide variant reduces the risk of sALS [360]. ALS-linked mutations in NEFH results in the accumulation of phosphorylated neurofilament proteins, which likely impairs axonal transport [361, 362]. Furthermore, the defective functioning of neurofilaments resulting from the mutations could contribute to neurofilamentous swellings in the axons of motor neurons [357].

**Peripherin (PRPH):** PRPH is a neuronal intermediate filament protein that regulates axonal transport [363]. Several PRPH mutations, generally resulting from aberrant splicing [364–366], are associated with ALS in humans, and overexpression of mutant PRPH in mice causes MN degeneration [367]. ALS-linked mutant. PRPH mutation disrupts the organization of neurofilament networks are a component of ubiquitinated soma and axonal inclusions in MNs of ALS patients [368, 369]. Existing evidence suggests, as in the case of NEFH, mutations in PRPH cause the formation of aggregate-containing inclusions and disrupt the cytoskeletal organization within MN axons impairing axonal transport of key cargos [370, 371]. Supporting the possibility that PRPH and NEFH contribute to common disease-promoting mechanisms is the finding that overexpression of NEFH and related neurofilament protein protect mice from PRPH-mediated MN death [372].

**Tubulin a-4A (TUBa4A / TUBA4A):** a-tubulins, including TUBA4A, are involved in regulating microtubule, stability, organization, and dynamics, disruption of which affects vesicular and axonal transport in neurons [373]. Mutations and variants in the TUBA4A gene encoding cause fALS [374–376]. Expression of ALS-associated TUBA4A mutants in MNs causes disintegration of microtubule organization and function [374]. Expression of TUBA4A is downregulated in the motor cortex of sALS patients and in fALS caused by mutation in other genes [377, 378], suggesting that fALS-causing TUBA4A mutations lead to a loss of function [373].

**DCTN1 (Dynactin-1):** DCTN is a multisubunit protein, enriched in neurons, that binds to both dynein motor proteins and microtubules, an interaction that is required for dynein-mediated retrograde axonal transport of vesicles and organelles [379–381]. The largest and key component of the DCTN complex is DCTN1, also referred to as p150^Glued^, which is the subunit that binds dynein [379–381]. Mutations in the DCTN1 gene cause ALS (as well as other MN degenerative diseases) to disrupt the association between DCTN and dynein, impairing dynein-mediated axonal transport of cargos including mitochondria and endosomes [180–182, 382, 383]. Mutations and other variations of DCTN1 are also linked to sALS [182]. Furthermore, DCTN1 expression is reduced in spinal cord MNs of ALS patients [384, 385]. One study has described the interaction between DCTN1 and TDP43 [386]. Expression of ALS-causing mutant DCTN1 results in mis localization and aggregation of TDP43. A recent study described that the reduction of DCTN1 in cultured results in the disassembly of stress granules and promotes TDP43 aggregation within inclusions [387].

**Profilin-1 (PFN1):** Through regulation of nucleotide exchange converting ADP-actin to ATP-actin, PFN1 plays a key role in the organization of the actin cytoskeleton, both inhibiting and promoting actin polymerization [388, 389]. Deregulation of actin polymerization and filament organization in MNs is a common feature in ALS and occurs before disease-related symptoms are observed [388, 390–392]. ALS-linked mutations in the PFN1 gene, which have been observed both in fALS and sALS, confer gain-of-function and result in progressive MN degeneration [392–396]. Mutant PFN1 also forms aggregates in inclusion containing p62, which sequester TDP43 [397–399]. These aggregates disrupt cytoskeleton organization affecting axonal transport and autophagy [397–399].

## Conclusions

In contrast to most other neurodegenerative diseases caused by mutations of a single gene, the development of fALS is linked to mutations or variants in many genes. How these structurally and functionally disparate groups of genes cause ALS remains unclear but may explain the pathophysiologic heterogeneity among patients with ALS. Additionally, the manner and the extent to which the multiple molecular and cellular abnormalities described in patients and experimental models of ALS contribute to disease pathogenesis have yet to be unresolved. Which of these are initiating factors and which follow the initiation of the disease process but also play a crucial role, is also unclear. In this review, we have focused on specific processes, including ER stress, vesicular transport, and axonal trafficking that are disrupted in ALS. Much attention has been placed on how mutant forms of the four major disease-causing genes are involved in the dysfunction of these specific processes. It is noteworthy that accumulation of protein aggregates as well as impairment in ER stress-coping mechanisms, vesicular transport, and axonal trafficking are prominent pathological features of many other age-associated neurodegenerative diseases but with distinct neurological symptoms. The commonalities and differences in the mechanisms between ALS and these other diseases concerning dysfunction of the processes on which this review focuses, remain to be clarified. Specifically, how distinct pathogenic triggers that cause different neurodegenerative diseases converge on common cellular abnormalities, such as vesicular or axonal transport is awaiting resolution. More information on these issues will lead to a better understanding of ALS (and other neurodegenerative disorders) and lead to the development of effective therapeutic approaches.

## Data Availability

No datasets were generated or analysed during the current study.
